# Novel reversibly switchable fluorescent proteins for RESOLFT and STED nanoscopy engineered from the bacterial photoreceptor YtvA

**DOI:** 10.1038/s41598-018-19947-1

**Published:** 2018-02-09

**Authors:** Carola Gregor, Sven C. Sidenstein, Martin Andresen, Steffen J. Sahl, Johann G. Danzl, Stefan W. Hell

**Affiliations:** 1 Department of NanoBiophotonics, Max Planck Institute for Biophysical Chemistry, Am Fassberg 11, 37077 Göttingen, Germany; 2 Max Planck Institute for Medical Research, Department of Optical Nanoscopy, Jahnstraße 29, 69120 Heidelberg, Germany; 3 Present Address: Abberior Instruments GmbH, Hans-Adolf-Krebs-Weg 1, 37077 Göttingen, Germany; 4 Present Address: Institute of Science and Technology Austria (IST Austria), Am Campus 1, 3400 Klosterneuburg, Austria

## Abstract

The reversibly switchable fluorescent proteins (RSFPs) commonly used for RESOLFT nanoscopy have been developed from fluorescent proteins of the GFP superfamily. These proteins are bright, but exhibit several drawbacks such as relatively large size, oxygen-dependence, sensitivity to low pH, and limited switching speed. Therefore, RSFPs from other origins with improved properties need to be explored. Here, we report the development of two RSFPs based on the LOV domain of the photoreceptor protein YtvA from *Bacillus subtilis*. LOV domains obtain their fluorescence by association with the abundant cellular cofactor flavin mononucleotide (FMN). Under illumination with blue and ultraviolet light, they undergo a photocycle, making these proteins inherently photoswitchable. Our first improved variant, rsLOV1, can be used for RESOLFT imaging, whereas rsLOV2 proved useful for STED nanoscopy of living cells with a resolution of down to 50 nm. In addition to their smaller size compared to GFP-related proteins (17 kDa instead of 27 kDa) and their usability at low pH, rsLOV1 and rsLOV2 exhibit faster switching kinetics, switching on and off 3 times faster than rsEGFP2, the fastest-switching RSFP reported to date. Therefore, LOV-domain-based RSFPs have potential for applications where the switching speed of GFP-based proteins is limiting.

## Introduction

The resolution of conventional fluorescence microscopes is limited by diffraction to $\frac{\lambda }{2\cdot NA}$, with *λ* denoting the emission wavelength and $NA$ the numerical aperture of the objective lens. Therefore, structures residing closer together than ~200 nm cannot be discerned using light in the visible range. However, several methods breaking the diffraction limit have been developed, notably STED (stimulated emission depletion) and RESOLFT (reversible saturable/switchable optical linear fluorescence transitions) nanoscopy with reversibly switchable fluorescent proteins (RSFPs), which allow imaging of living cells with higher resolution. STED nanoscopy uses stimulated emission to reversibly silence fluorophores located in the outer regions of a diffraction-limited spot of excitation light, thereby decreasing the region from which fluorescence is possible. Since stimulated emission has to occur before spontaneous emission of fluorescence, relatively high STED intensities are required as the lifetime of the excited state is typically in the range of only a few nanoseconds. The light intensity required to switch molecules into a dark state can be decreased if the on- and the off-state are long-lived. This is the basis for RESOLFT microscopy, which applies the switching of fluorescent proteins between two different conformations, one of which is non-fluorescent^1^.

**Figure 1:**
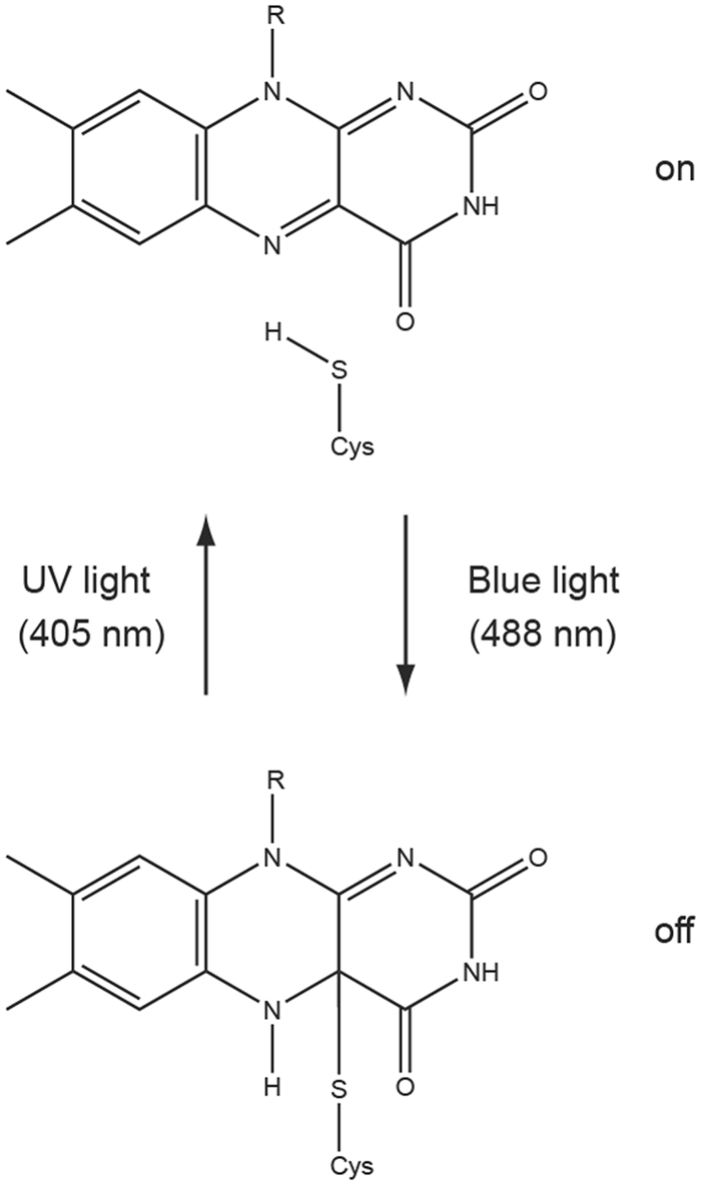
Photoswitching of YtvA-LOV. Before illumination, YtvA-LOV resides in a fluorescent state (on, top) in which the FMN cofactor is non-covalently bound to the protein, of which one amino acid (Cys-SH) is shown. Absorption of blue light switches the protein into a non-fluorescent state (off, bottom) by formation of a covalent bond between the FMN and the cysteine residue (Cys) of the protein. The fluorescent state can be partially reconstituted with UV light. R: ribityl side chain.

While STED nanoscopy requires relatively strong light intensities to achieve the highest resolutions, the lower-light-level RESOLFT methods are limited particularly by the attainable imaging speed determined by the switching kinetics of the RSFP. This poses a problem for the observation of quickly moving structures in living cells. For this purpose, GFP-related RSFPs^2–7^ have been developed that are optimized for fast switching^5,6^, achieving pixel dwell times during imaging of down to 75 µs^5,8^. All commonly used RSFPs are derived from GFP-related proteins and share several features that limit their applicability as fluorescent reporter proteins. First, their relatively large size of 27 kDa can affect the function of fusion proteins and therefore produce artifacts. Second, GFP fluorescence is strongly quenched at low pH, impeding the imaging of acidic organelles such as lysosomes. Third, GFP fluorescence is dependent on oxygen for chromophore maturation and can therefore not be used under anaerobic conditions. FMN (flavin mononucleotide)-binding fluorescent proteins (FbFPs) overcome all these limitations. These proteins are smaller (12–19 kDa^9^) and their fluorescence is oxygen-independent and less sensitive to low pH^10,11^. Several FbFPs were engineered on the basis of different LOV (light-oxygen-voltage sensing) domains^11–14^. LOV apoproteins are non-fluorescent, but under cellular conditions they acquire fluorescence by non-covalent binding of FMN, a fluorescent cofactor that is abundant in all cell types. Upon absorption of blue light, LOV domains undergo a photocycle that leads to formation of a covalent bond between the FMN and a cysteine residue of the protein, thereby switching the protein into a non-fluorescent state (Fig. 1). After seconds to hours, the photoadduct decays back into the non-covalently bound, fluorescent state. Cleavage of the photoadduct can be accelerated by UV light, but this reconstitutes only ~10% of the initial fluorescence since UV light also triggers the forward, off-switching photoreaction, resulting in an equilibrium of both states^15^. To make LOV domains constitutively fluorescent with emission in the green range, their photocycle was abolished by mutating the photoactive cysteine residue into alanine^11^. In addition, their brightness and photostability were further improved by mutagenesis^12,13^. On the other hand, their inherent photoswitching makes LOV domains interesting for nanoscopy (superresolution microscopy). This has been demonstrated with the LOV domain from the photoreceptor protein YtvA (YtvA-LOV) from *Bacillus subtilis* in PALM imaging^15^. In this study^15^, the wild-type LOV domain was used, which has a relatively weak fluorescence and switches back to the fluorescent state under UV light to less than 10%. This hampers its use for RESOLFT applications, where multiple switching cycles have to be performed. We aimed at improving the switching and brightness of YtvA-LOV by site-directed and error-prone mutagenesis. This resulted in the improved reversibly switchable protein rsLOV1 that exhibits an effective brightness ~10-fold higher compared to the wild-type protein under RESOLFT imaging conditions due to improved on-switching. In addition, we generated a second variant rsLOV2 with an even ~2-fold further increased fluorescence that was used for STED imaging.

## Results

**Figure 2:**
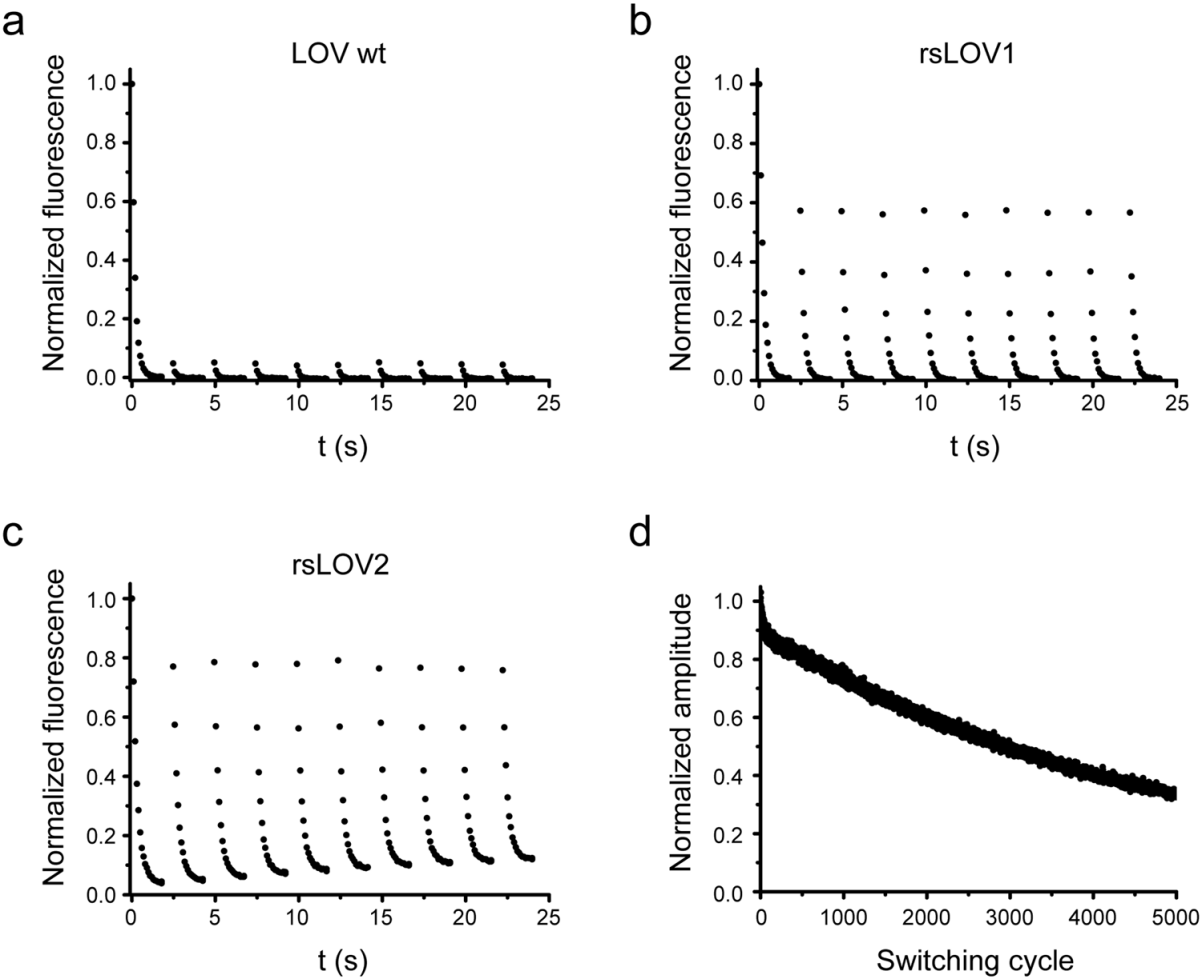
Switching of YtvA-LOV wt, rsLOV1, and rsLOV2 under low-power screening conditions. (**a**–**c**) Purified proteins in solution were switched off with 488 nm light (~2 W∙cm^−2^) for 2 s and switched on with 405 nm light (~5 W∙cm^−2^) for 0.5 s. For better clarity, only off-switching is shown. The fluorescence signal was normalized to the initial value. Data are representative of three independent measurements. (**d**) Purified rsLOV1 in solution was switched off with 488 nm light for 2 s (~2 W∙cm^−2^) and switched on with 405 nm light (~5 W∙cm^−2^) for 0.5 s 5000 times. Off-switching curves were fitted with a single exponential decay function. The normalized amplitudes of the fits are displayed. The first switching cycle was omitted. Data are representative of three independent measurements.

To improve the brightness of YtvA-LOV upon 488 nm excitation after repeated on- and off-switching with 405 and 488 nm light, respectively, multiple rounds of site-directed and error-prone mutagenesis were performed. Colonies of *E. coli* cells expressing YtvA-LOV variants were grown at 37 °C and screened for increased off-switching amplitude after repeated RESOLFT-type on/off-switching (here referred to as RESOLFT brightness RB) with an automated microscope. Multiple mutations were identified that contributed to enhanced RB by improved on-switching and possibly also by better expression and folding at 37 °C. Two variants, named rsLOV1 and rsLOV2 (reversibly switchable LOV1 and 2), were obtained that contain the mutations indicated in Supplementary Fig. S1. rsLOV1 exhibits a similar off-switching amplitude as the wild-type LOV domain (LOV wt) in the first switching cycle. However, upon irradiation with UV light (405 nm), 57% of the initial signal is recovered, compared to ~5% for LOV wt under our screening conditions (Fig. 2a–c). This corresponds to a ~10-fold increase in RB. The switching background of rsLOV1 during the switching cycle is less than 3%, indicating an almost complete off-switching with 488 nm light. rsLOV2 exhibits an initial signal ~2 times higher than LOV wt in the first cycle and shows an even better on-switching to 78% of the initial value, increasing the RB ~30-fold compared to the wild-type protein. However, the switching background of rsLOV2 increases after repeated switching (Fig. 2a–c), an undesirable feature for RESOLFT applications.

Since photostability of RSFPs upon repeated switching is an important feature for RESOLFT applications, we measured 5000 switching cycles of rsLOV1 under the low-power conditions (few W·cm^−2^) used for screening. rsLOV1 retained 50% of its RB after 3000 switching cycles (Fig. 2d). Under high-power excitation comparable to RESOLFT imaging conditions, however, rsLOV1 lost 50% of its RB within the first 10 switching cycles (Supplementary Fig. S2). This loss of signal was reversible and was almost fully reconstituted after a break of 1 min, possibly indicating the transition to a further, alternate long-lived dark state. Similar reversible photobleaching was observed in rsLOV2 as well (Supplementary Fig. S2) and has also been described for the LOV domain-based fluorescent protein iLOV^12^, where the photocycle is abolished.

**Figure 3:**
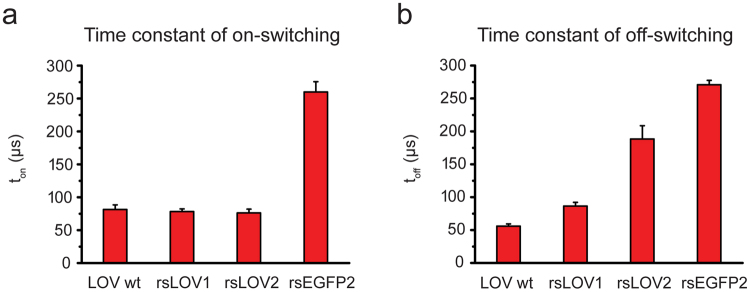
Time constants of on- and off-switching of YtvA-LOV wt, rsLOV1, rsLOV2, and rsEGFP2. Measurements were performed with purified proteins under high-power conditions (on-switching: 405 nm, ~100 kW∙cm^−2^, off-switching: 488 nm, ~300 kW∙cm^−2^). (**a**) On- and (**b**) off-switching curves were fitted with a single exponential function (Supplementary Fig. S3). Error bars represent the standard deviation of the mean (n = 15). Data are representative of three independent measurement series.

Next, we compared the switching kinetics of rsLOV1 and rsLOV2 as well as the wild-type protein LOV wt to rsEGFP2^5^, the fastest switching RSFP published so far. On- and off-switching were performed at 405 and 488 nm, respectively, with the same powers for all proteins. LOV wt, rsLOV1, and rsLOV2 switched on ~3 times faster than rsEGFP2 (Fig. 3a). The on-switching time constant of rsLOV1 and rsLOV2 was not significantly affected by mutagenesis. LOV wt switched off 5 times faster than rsEGFP2 (Fig. 3b). Off-switching of rsLOV1 and rsLOV2 was slower, but still faster than rsEGFP2 by a factor of 3 and 1.4, respectively. Since the duration of the overall switching cycle of rsLOV1 is reduced by a factor of 3 compared to rsEGFP2, rsLOV1 appears interesting for fast RESOLFT imaging.

**Figure 4:**
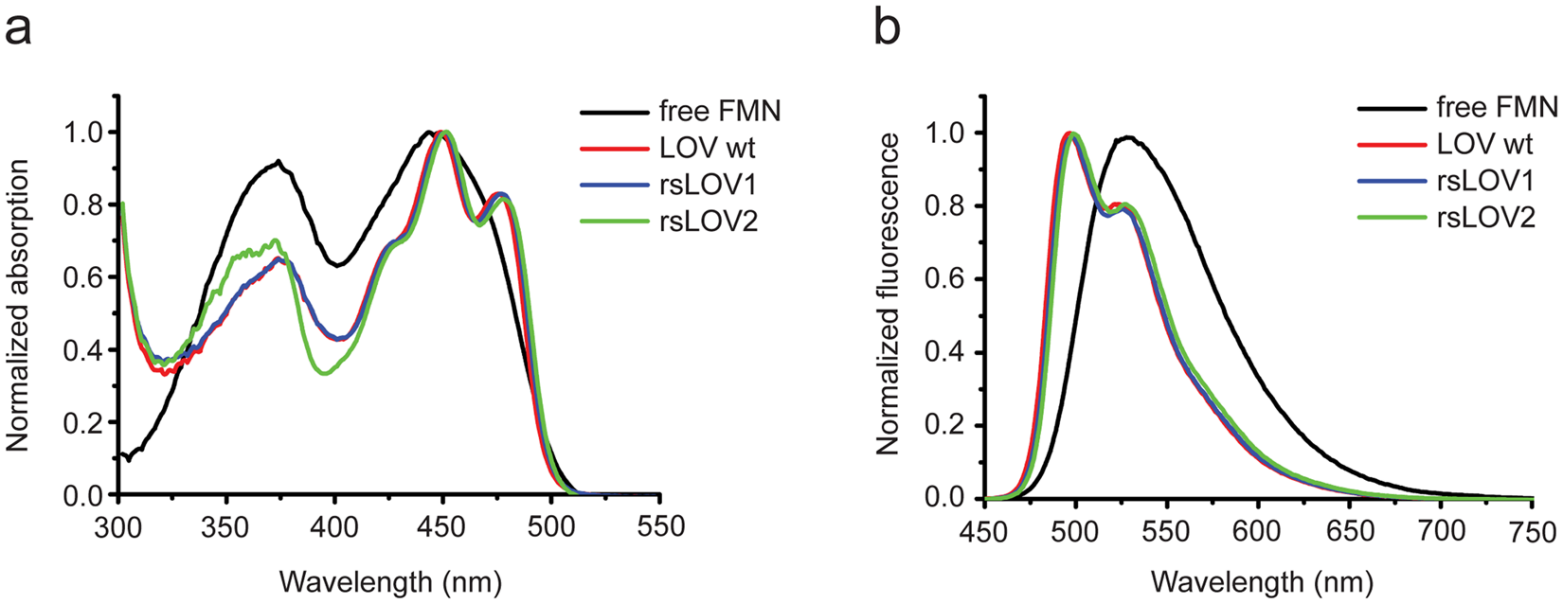
Absorption and fluorescence emission spectra of FMN, LOV wt, rsLOV1, and rsLOV2. (**a**) Normalized absorption spectra of the free FMN used as a reference, as well as the FMN-loaded proteins LOV wt, rsLOV1, and rsLOV2. (**b**) Normalized fluorescence emission spectra of FMN, LOV wt, rsLOV1, and rsLOV2 excited at 440 nm.

We measured spectra of purified GST-tagged LOV wt, rsLOV1, and rsLOV2 (Fig. 4). The spectra of rsLOV1 and rsLOV2 were similar to the wild-type protein with absorption and fluorescence emission maxima of ~450 and 498 nm, respectively. Extinction coefficients ε were calculated from the absorption at 450 nm (Table 1), with the assumption that the proteins are fully saturated with FMN. Therefore, the given values represent the lower limit for ε. Quantum yields were determined using FMN as a reference (ɸ_fl_ = 0.246, ref.^16^). Since the protein solution was not illuminated prior to the measurement, the values given in Table 1 correspond to the fluorescent on-state brightness (i.e., the initial signal of the first off-switching cycle). Our data indicate that the extinction coefficients of rsLOV1 and rsLOV2 are not significantly altered by mutagenesis. In addition, the quantum yield of rsLOV1 is identical to that of LOV wt, demonstrating that its improved RB is solely due to improved on-switching. rsLOV2 exhibits a quantum yield almost 2 times higher than LOV wt, consistent with the amplitudes measured during the first cycle of off-switching. These results demonstrate that the on-state brightness (given by the product of extinction coefficient and quantum yield) of rsLOV1 and rsLOV2 is still low compared to EGFP (6% and 11% for rsLOV1 and rsLOV2, respectively, see Table 1). Compared to rsEGFP2, the on-state brightness of rsLOV1 and rsLOV2 is 10% and 19%, respectively.

**Table 1:** Extinction coefficients and fluorescence quantum yields of LOV wt, rsLOV1, and rsLOV2.

	ε [M^−1^·cm^−1^]	ɸ_fl_
free FMN	12,200 (ref.^22^)	0.25 (ref.^16^)
LOV wt	12,700	0.17
rsLOV1	10,900	0.17
rsLOV2	11,400	0.31
EGFP	55,000 (ref.^23^)	0.60 (ref.^24^)
rsEGFP2	61,300 (ref.^5^)	0.30 (ref.^5^)

The numbers for extinction coefficients represent values at 450 nm for FMN, LOV wt, rsLOV1, and rsLOV2, at 488 nm for EGFP, and at 478 nm for rsEGFP2.

Wild-type YtvA-LOV (aa 25–126) has been reported to dimerize, whereas dimerization is prevented in full-length YtvA^17^. We observed that GST-tagged YtvA-LOV (aa 1–146), rsLOV1, and rsLOV2 migrate mostly as monomers on semi-native gels (Supplementary Fig. S4). To test the behavior of rsLOV in mammalian cells, we expressed a fusion protein of rsLOV2 and histone H2B which requires a monomeric tag for proper localization (Supplementary Fig. S5). We observed that localization of H2B is not impaired by the tag, indicating that rsLOV2 is predominantly monomeric under cellular conditions. rsLOV2 was also used for labeling of other cellular structures (Supplementary Fig. S5), demonstrating its utility as a protein tag in living cells. Since its on-state brightness is relatively low, autofluorescence from lysosomes around the nucleus is clearly visible in some cells.

**Figure 5:**
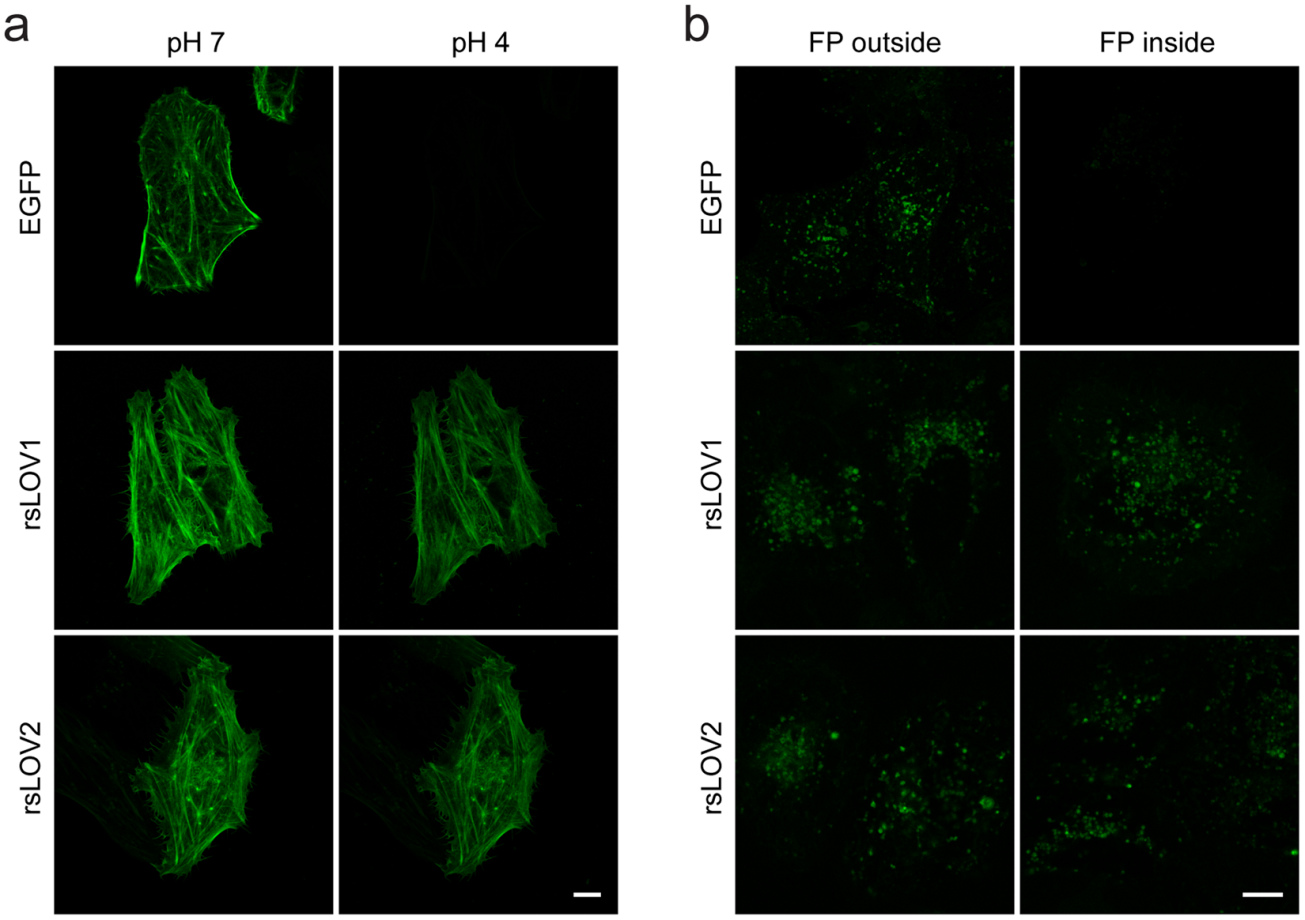
Fluorescence of EGFP, rsLOV1, and rsLOV2 at low pH. (**a**) CV-1 cells expressing lifeact-EGFP, lifeact-rsLOV1, and lifeact-rsLOV2 were permeabilized with 1 µM nigericin in a citric acid/phosphate buffer of pH 7 and 4 and imaged with a confocal microscope. Images at pH 7 and 4, respectively, were acquired with identical settings and colormap. Images are representative of three different cells. (**b**) Confocal images of HeLa cells expressing LAMP1 tagged with EGFP, rsLOV1, and rsLOV2 at the inner or outer side of the lysosomal membrane. Images with the fluorescent protein (FP) at the outside and inside, respectively, were acquired with identical settings and colormap. Images are representative of ten different cells. Scale bars: 10 µm.

The fluorescence of LOV domains from different origin has been found to be insensitive to changes in pH over a broad range^10^. This makes LOV domains superior for imaging at low pH where GFP fluorescence is strongly quenched. To test if this feature is preserved in rsLOV1 and rsLOV2, we measured switching curves of purified proteins in citric acid/phosphate buffers of different pH values (Supplementary Fig. S6). Whereas rsEGFP2 did not yield sufficient fluorescence signal for fitting of the switching curves below pH 6, rsLOV1 and rsLOV2 retained 38 and 73% of their fluorescence down to pH 4 compared to pH 7. EGFP fluorescence also strongly decreased with pH and was not detectable at pH 4. We subsequently imaged rsLOV1 and rsLOV2 in acidic surroundings within living cells. First, we permeabilized CV-1 cells expressing EGFP, rsLOV1, and rsLOV2 fused to the actin-binding peptide lifeact^18^ with nigericin in citric acid/phosphate buffer at pH 7 and 4 (Fig. 5a). As expected, EGFP fluorescence is strongly reduced at low pH whereas fluorescence from rsLOV1 and rsLOV2 is largely preserved at pH 4. Therefore, rsLOV1 and rsLOV2 should be superior to EGFP for imaging of acidic organelles such as lysosomes (pH 4.5–5.0, ref.^19^). We labeled the lysosomal transmembrane protein LAMP1 (lysosomal-associated membrane protein 1) with EGFP, rsLOV1, and rsLOV2 at its C-terminal end which resides in the cytoplasm, as well as N-terminally of the transmembrane domain to target the fluorescent protein to the interior of the lysosome. As expected, fluorescence of EGFP inside the vesicles is strongly quenched in comparison to the cytosolic side of the membrane, whereas fluorescence of rsLOV1 and rsLOV2 is mostly preserved (Fig. 5b). This confirms the suitability of rsLOV1 and 2 to image acidic organelles in living cells.

**Figure 6:**
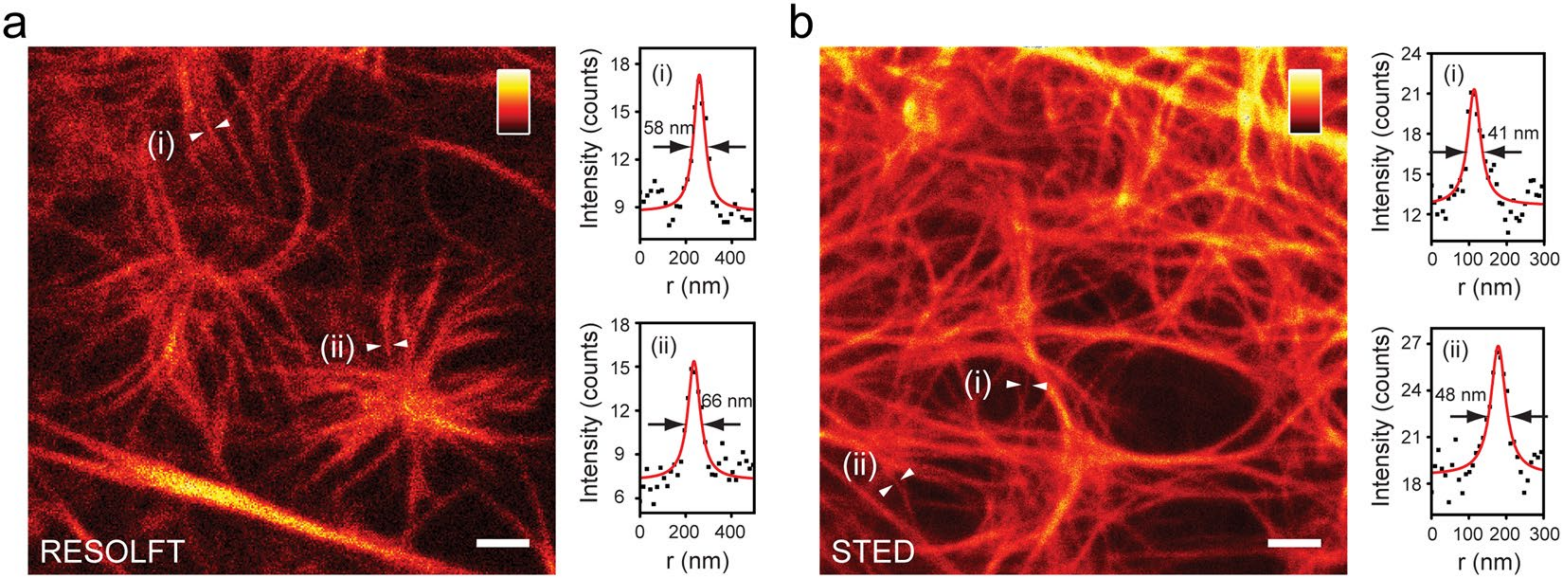
Live-cell nanoscopy with rsLOV1 and rsLOV2. (**a**) RESOLFT image of lifeact-rsLOV1. Living CV-1 cells expressing lifeact-rsLOV1 were imaged with the following pulse sequence: 10 µs activation (405 nm confocal), 1 µs break, 200 µs deactivation (488 nm donut), 1 µs break, 5 µs readout (488 nm confocal). The signal from 20 scans per line was accumulated. FWHM values of (i) and (ii) are 58 nm and 66 nm, respectively. (**b**) STED image of lifeact-rsLOV2. Living CV-1 cells expressing lifeact-rsLOV2 were imaged with 488 nm excitation (pulsed) and 618 nm STED (pulsed) with a pixel dwell time of 200 µs. The signal from 3 scans per line was accumulated. FWHM values of (i) and (ii) are 41 nm and 48 nm, respectively. Images are representative of 10 different cells. Images represent raw data. Line profiles were fitted with a Lorentzian function. Scale bars (**a,b**): 1 µm.

We used rsLOV1 for RESOLFT imaging of actin in living CV-1 cells (Fig. 6a). For each pixel, the proteins were switched on with 405 nm light for 10 µs, switched off with a 488 nm donut for 200 µs, and read out with a confocal 488 nm beam for 5 µs. To obtain sufficient signal, 20 scans per line were accumulated. The thinnest feature widths that can be robustly extracted (60–70 nm FWHM, Fig. 6a) from the RESOLFT recording suggest a resolution level in the range of 70–80 nm. The resolution seems to be limited by the low RESOLFT brightness of rsLOV1, as thinner and therefore darker filaments do not provide sufficient signal above background. Interestingly, the second protein rsLOV2 proved to be useful for STED imaging (Fig. 6b). Apart from the reversible photobleaching, the protein appeared to be very photostable even at high STED intensities (Supplementary Fig. S7). We observed that bleaching was reduced when omitting the 405 nm on-switching pulse and that imaging could be performed solely with 488 nm excitation and simultaneous 618 nm STED. Lines were scanned 3 times with a pixel dwell time of 200 µs. The thinnest robust features of 40–50 nm (Fig. 6b) indicate a resolution level of down to 50–60 nm.

## Discussion

The potential of wild-type YtvA-LOV for PALM imaging has already been outlined^15^, but its low on-state brightness in combination with inefficient on-switching hinders its use for RESOLFT microscopy. In this work, we demonstrated that the on-switching of YtvA-LOV by 405 nm light can be improved 10-fold by mutagenesis, resulting in the new protein rsLOV1 that can be used for RESOLFT imaging of living cells with a resolution of down to 70–80 nm. An interesting feature of rsLOV1 is its very fast on- and off-switching, pointing to its potential for faster RESOLFT imaging than has been achieved with GFP-related RSFPs so far. Yet, the low number of photons per switching cycle of rsLOV1 required repeated scanning of the sample to achieve sufficient image contrast (by collecting sufficient signal), thereby reducing the speed of imaging. Hence, further improvements in the RESOLFT brightness of rsLOV1 are needed. This could be achieved either by further changes in the protein sequence or by exchanging its cofactor FMN against flavins with higher extinction coefficients and quantum yields, as has been described *in vitro*^20,21^. However, the modified cofactor would need to be incorporated into the protein *in vivo* and is likely to influence the switching behavior. Therefore, further mutagenesis and screening in the presence of the exchanged cofactor might be necessary for improving the performance further.

The second variant rsLOV2 is brighter than rsLOV1, but exhibits a significant switching background that hinders its application for RESOLFT imaging. We showed, however, that rsLOV2 can be used for STED imaging with a resolution of down to 50–60 nm despite its faster apparent photobleaching under low-power screening conditions. Interestingly, rsLOV2 exhibited very little photobleaching even at high STED intensities at 618 nm (38 mW power in the back aperture) when excited at 488 nm. Yet, prior or simultaneous irradiation with 405 nm light led to a fast decrease in fluorescence even during confocal imaging. Surprisingly, STED imaging with 488 nm excitation without prior 405-nm on-switching turned out to be superior for imaging. A possible explanation for this is that at least the initial on-switching can be accomplished by (dual-colour) two-photon absorption facilitated by the STED beam (at the prior scan coordinate). The fluorescence process could also occur after transitioning through other states. The involvement of other states may also explain the observation that, for a substantial range of pixel dwell times (up to hundreds of microseconds), longer dwell times allowed to accumulate ever more signal – despite the 488 nm laser, which should transfer rsLOV2 to its off-state, acting at the doughnut center (targeted coordinate) all this time.

By optimization of non-switchable FbFP variants with the specific STED requirements in mind, it may be possible in future work to generate even brighter and more photostable proteins that would supplement the toolbox of fluorescent proteins for superresolution imaging of living cells.

## Materials and Methods

### Mutagenesis and Screening

The LOV domain of YtvA including the C-terminal linker (aa 1–146) was cloned into the vector pGEX-6 P-1 between the BamHI and SalI restriction sites. Error-prone PCRs were performed with the primers TTAGTCGGATCCATGGCTAGTTTTCAATCA and TTAGTCGTCGACTTAAAGTGCAGTAATTTC with 25 PCR cycles and MnCl_2_ concentrations ranging from 100 to 300 µM. Screening was performed with the expression vector pGEX-6 P-1 in *E. coli* DH5α cells. Cells were grown on LB agar plates over night at 37 °C and screened at room temperature. Screening was performed with an automated microscope under low-power conditions with 405 nm (~2 W∙cm^−2^) and 488 nm (~5 W∙cm^−2^) for on- and off-switching, respectively.

### Protein purification and characterization

GST-tagged LOV wt, rsLOV1, rsLOV2, and EGFP were expressed from the vector pGEX-6 P-1 in *E. coli* SURE cells. Cells were grown at 37 °C to an OD_600_ between 0.5 and 0.6 and induced with 1 mM IPTG (AppliChem) over night at 30 °C. Cells were lysed in B-PER solution (Thermo Scientific) and purified by affinity chromatography with glutathione spin columns (Thermo Scientific) according to the instructions of the manufacturer. The eluted protein was ultrafiltrated against 150 mM NaCl, 50 mM Tris-HCl, pH 8.0. Unless otherwise indicated, all measurements with purified proteins were performed in this buffer. Protein concentration was determined by Bradford assay. Fluorescence measurements under low-power conditions were performed with an automated microscope with approximate intensities of 5 W∙cm^−2^ for 405 nm and 2 W∙cm^−2^ for 488 nm using 3 µl of protein solution. Intensities under high-power conditions were approximately 100 kW∙cm^−2^ for 405 nm and 300 kW∙cm^−2^ for 488 nm. Gel electrophoresis was performed with 15% acrylamide gels containing 0.1% SDS. For denaturing gels, samples were boiled for 2 min at 99 °C in a loading buffer containing 100 mM DTT and 2% (w/v) SDS. For seminative gels, samples were applied to the gel in a loading buffer without DTT and SDS without previous heating.

Absorption and fluorescence emission spectra were recorded with a Varian Cary 4000 UV/VIS spectrophotometer and a Varian Cary Eclipse fluorescence spectrophotometer, respectively. Measurements were performed without previous illumination of the proteins in order to keep the proteins in the fluorescent on-state. Extinction coefficients were calculated from the absorption at 450 nm and the protein concentrations determined by the Bradford assay, assuming that the proteins are fully saturated with FMN. Quantum yields of GST-tagged LOV wt, rsLOV1, and rsLOV2 were determined using FMN (HPLC-purified, purity ≥ 95%, Sigma-Aldrich) as a reference, assuming a quantum yield for FMN of 0.246, ref.^16^).

### Cloning

Codon-optimized versions of rsLOV1, rsLOV2, and EGFP for expression in mammalian cells were cloned into the given vectors with the primers and restriction enzymes indicated in Table 2. Lifeact, MAP2, LAMP1 (FP C-terminal), and H2B were integrated into pcDNA3.1(+) between the following restriction sites: lifeact: HindIII/BamHI, MAP2: AscI/NotI, LAMP1: HindIII/BamHI, H2B: NheI/HindIII. Vimentin-rsLOV2 was expressed from the vector pmKate2 where vimentin was integrated between the EcoRI and AgeI restriction sites. LAMP1 containing rsLOV1, rsLOV2, or EGFP in front of the transmembrane domain was constructed in the following way: the N-terminal fragment of LAMP1 was amplified with the primers LAMP1 Nt HindIII fwd/LAMP1 Nt BamHI rev and cloned into pcDNA3.1(+) with HindIII and BamHI. Subsequently, the C-terminal fragment of LAMP1 including the transmembrane domain was amplified with the primers LAMP1 Ct EcoRI fwd/LAMP1 Ct NotI rev and cloned between the EcoRI and NotI restriction site. rsLOV1, rsLOV2, and EGFP were amplified with the primers given in Table 2 and integrated between the BamHI and EcoRI restriction sites.

**Table 2:** Primers used for cloning of lifeact, MAP2, LAMP1, H2B, and vimentin constructs of rsLOV1 and rsLOV2.

Construct	Primer	Sequence
lifeact	rsLOV BamHI fwd	GTTGATGGATCCACCGGTCGCCACCATGACAAGATTTCAGTCA
	rsLOV NotI rev	TTGATCGCGGCCGCTCAGCCTCTGTGCGGTCTCTC
	EGFP BamHI fwd	TTATCAGGATCCACCGGTCGCCACCGTGAGCAAGGGCGAG
	EGFP NotI rev	GTCGCGGCCGCTACTTGTACAGCTCGTCCATGCCGAGAG
MAP2	rsLOV NheI fwd	AAGCTGGCTAGCATGACAAGATTTCAGTCA
	rsLOV AscI rev	GATCCTGGCGCGCCGCCTCTGTGCGGTCTCTC
LAMP1 (FP C-terminal)	rsLOV EcoRI fwd	GTGGTGGAATTCCATGACAAGATTTCAGTCA
	rsLOV XhoI rev	TCTAGACTCGAGTCAGCCTCTGTGCGGTCTCTC
	EGFP EcoRI fwd	CAGAGAATTCCATGGTGAGCAAGGGCGAGGAGC
	EGFP XhoI rev	CGTACTCGAGTTACTTGTACAGCTC
H2B	rsLOV BamHI fwd	GAATCAGGATCCATGACAAGATTTCAGTCA
	rsLOV XbaI rev	ATCAACTCTAGATCAGCCTCTGTGCGGTCTCTC
vimentin	rsLOV AgeI fwd	GTAGATACCGGTCGCCACCATGACAAGATTTCAGTCA
	rsLOV NotI rev	TTGATCGCGGCCGCTCAGCCTCTGTGCGGTCTCTC
LAMP1 with FP in front of TMD	LAMP1 Nt HindIII fwd	TCGATCAAGCTTATGGCGGCCCCCGGCAGC
	LAMP1 Nt BamHI rev	TTAGAAGGATCCACTACCGCTCAGCATGCTGTTCTCGTC
	LAMP1 Ct EcoRI fwd	TAGTATGAATTCAGCGGCTCTATCCCCATCGCTGTGGGT
	LAMP1 Ct NotI rev	TCGATCGCGGCCGCCTAGATAGTCTGGTAGCCTGC
	rsLOV BamHI fwd	GAATCAGGATCCATGACAAGATTTCAGTCA
	rsLOV EcoRI rev	GCTTGTGAATTCGCCTCTGTGCGGTCTCTC
	EGFP BamHI fwd	TACGGATCCATGGTGAGCAAGGGCGAGGAG
	EGFP EcoRI rev	TAGAGAATTCCTTGTACAGCTCGTCCATGCCGAGAG

### Mammalian cell culture

CV-1 and HeLa cells were grown in DMEM with 4.5 g/l glucose (Gibco) supplemented with 10% FBS, 1 mM sodium pyruvate, 100 units/ml penicillin, and 100 µg/ml streptomycin (all Biochrom). Cells were cultured at 37 °C with 5% CO_2_. CV-1 and HeLa cells were transfected with Lipofectamine 2000 (Invitrogen) according to the protocol of the manufacturer and imaged the following day.

### Imaging

All images were recorded in living cells. Confocal images were taken with a Leica SP8 microscope with simultaneous excitation at 405 and 488 nm for rsLOV1 and rsLOV2. EGFP fluorescence was excited at 488 nm. Fluorescence was recorded between 495 and 600 nm for all constructs. For measurements at different pH, CV-1 cells were permeabilized with 1 µM nigericin (Sigma Aldrich) in a citric acid/phosphate buffer of pH 7 or 4 supplemented with 140 mM KCl (Sigma Aldrich).

RESOLFT and STED images were taken with a home-built setup described in ref.^8^. The STED laser was exchanged to a fibre laser with a repetition rate of 40 MHz and a wavelength of 618 nm (MPB Communications Inc.). The following powers were used for RESOLFT imaging (measured in the back aperture): 405 nm activation (confocal): 5.1 µW (CW), 488 nm off-switching (donut): 4.8 µW (CW), 488 nm readout (confocal): 4.2 µW (pulsed, repetition rate 40 MHz). Imaging was performed with the following pulse sequence: 10 µs activation (405 nm), 1 µs break, 200 µs deactivation (488 nm donut), 1 µs break, 5 µs readout (488 nm confocal). The signal from 20 scans per line was accumulated. The pixel size was 30 nm. The image represents raw data.

For STED imaging, the following powers were used (measured in the back aperture): 488 nm excitation (confocal): 3.0 µW, 618 nm STED (donut): 38 mW (both lasers pulsed, repetition rate 40 MHz). Pixel dwell time was 200 µs. Nanosecond time gating was used. The signal from 3 scans per line was accumulated. The pixel size was 30 nm for confocal and 15 nm for STED images. Images represent raw data.

### Data Analysis

To determine switching time constants and amplitudes, switching curves were fitted with Matlab (MathWorks) or OriginPro software (OriginLab) using the single exponential function $y=y0+A\cdot {e}^{-\frac{t}{\tau }}$ with *t* denoting time, *y* the fluorescence signal, *y*0 the switching background, *A* the amplitude, and τ the switching time constant.

Resolution of RESOLFT and STED images was determined by the FMWH (full width at half maximum) of line profiles with a width of 5–10 pixels. The signal was fitted with a Lorentzian function $y=y0+\frac{2\cdot A}{\pi }\cdot (\,\frac{w}{4{(x-{x}_{C})}^{2}}+{w}^{2})$ with *w* denoting the FWMH, using OriginPro software (OriginLab).

## Electronic supplementary material

Supplementary information
