# Supplementary material for: Novel reversibly switchable fluorescent proteins for RESOLFT and STED nanoscopy engineered from the bacterial photoreceptor YtvA
Source: Sci Rep. 2018 Feb 9;8:2724. doi: 10.1038/s41598-018-19947-1 (PMC5807511; doi:10.1038/s41598-018-19947-1)
Supplement: Supplementary file 1 — Supplementary information [file 41598_2018_19947_MOESM1_ESM.pdf]

Supplementary Information

Novel reversibly switchable fluorescent proteins for RESOLFT and STED nanoscopy engineered from the bacterial photoreceptor YtvA

Carola Gregor[1,*], Sven C. Sidenstein[1,2], Martin Andresen[1], Steffen J. Sahl[1], Johann G. Danzl[1,3] & Stefan W. Hell[1,4,*]

[1]Max Planck Institute for Biophysical Chemistry, Department of NanoBiophotonics, Am Fassberg 11, 37077 Göttingen, Germany
[2]Current address: Abberior Instruments GmbH, Hans-Adolf-Krebs-Weg 1, 37077 Göttingen, Germany
[3]Current address: Institute of Science and Technology Austria (IST Austria), Am Campus 1, 3400 Klosterneuburg, Austria
[4]Max Planck Institute for Medical Research, Department of Optical Nanoscopy, Jahnstraße 29, 69120 Heidelberg, Germany

* Correspondence should be addressed to S.W.H (email: shell@gwdg.de) or C.G. (email: cgregor@gwdg.de).

Contents

Supplementary Figure S1
Supplementary Figure S2
Supplementary Figure S3
Supplementary Figure S4
Supplementary Figure S5
Supplementary Figure S6
Supplementary Figure S7

```
LOV wt     MASFQSFGIPGQLEVIKKALDHVRVGVVITDPALEDNPIVYVNQGFVQMTGYETEEILGK
rsLOV1     MTRFQSFGISGQLEVIKNALDHLRVGVVITDPAREDNPIVYVNQGFVQMTGYEAEEILGK
rsLOV2     MTRFQSFGISGQLEVIKNALDHLRVGVVITDPAREDNPVVYVNNGFVQMTGYEAEEILGK

LOV wt     NCRFLQGKHTDPAEVDNIRTALQNKEPVTVQIQNYKKDGTMFWNELNIDPMEIEDKTYFV
rsLOV1     NCRILQGEHTDPAEVDIIRTALQNKEPVTVQILNYRKDGTMFWNLLHIVPIVIEGKTYFV
rsLOV2     SCRILQGEHTDPAEVDIIRTALQNKEPVTVQILNYRKDGTMFWNLLHIVPIVIEGKTYFV

LOV wt     GIQNDITKQKEYEKLLEDSLTEITAL
rsLOV1     GNQNDITKQKEYEKLLERPHRG----
rsLOV2     GNQNDITKQKEYEKLLERPHRG----
```

Supplementary Figure S1. Sequence alignment of YtvA-LOV wt (aa 1–146), rsLOV1, and rsLOV2. rsLOV1 and rsLOV2 consist of the first 137 amino acids of YtvA with the indicated mutations and have a deviating C-terminal extension. Mutations in rsLOV1 and rsLOV2 are highlighted in green.

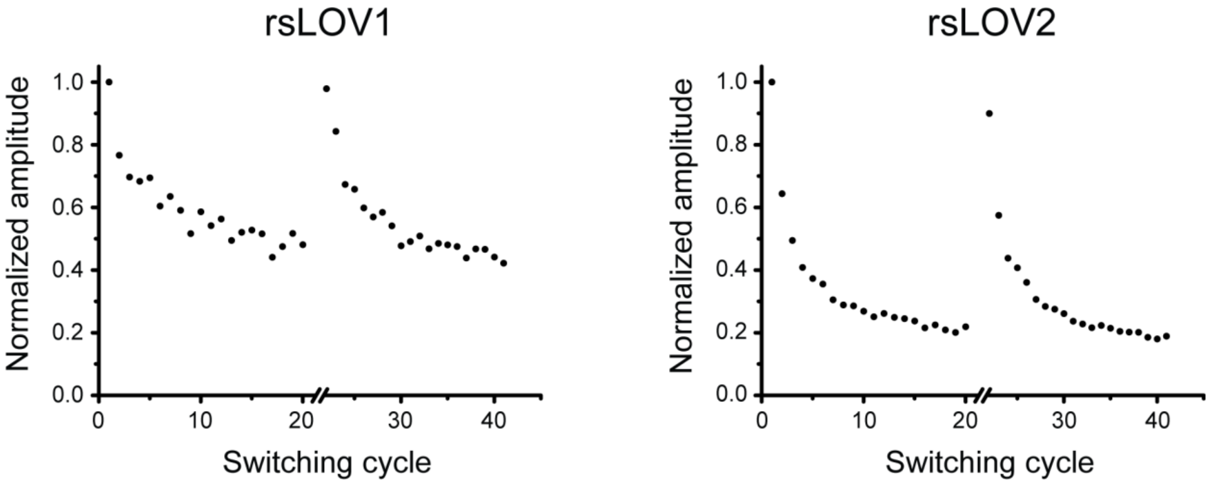

Supplementary Figure S2. Fast on-state signal reduction (“bleaching“) of rsLOV1 and rsLOV2 under high-power conditions is reversible. 40 switching cycles of rsLOV1 and rsLOV2 were recorded under high-power conditions (~100 kW·cm^{-2} for 405 nm and ~300 kW·cm^{-2} for 488 nm) with a break of 1 min after the first 20 switching cycles. Normalized off-switching amplitudes fitted with a single exponential function are shown. Measurements were performed in *E. coli* SURE colonies to rule out diffusional effects.

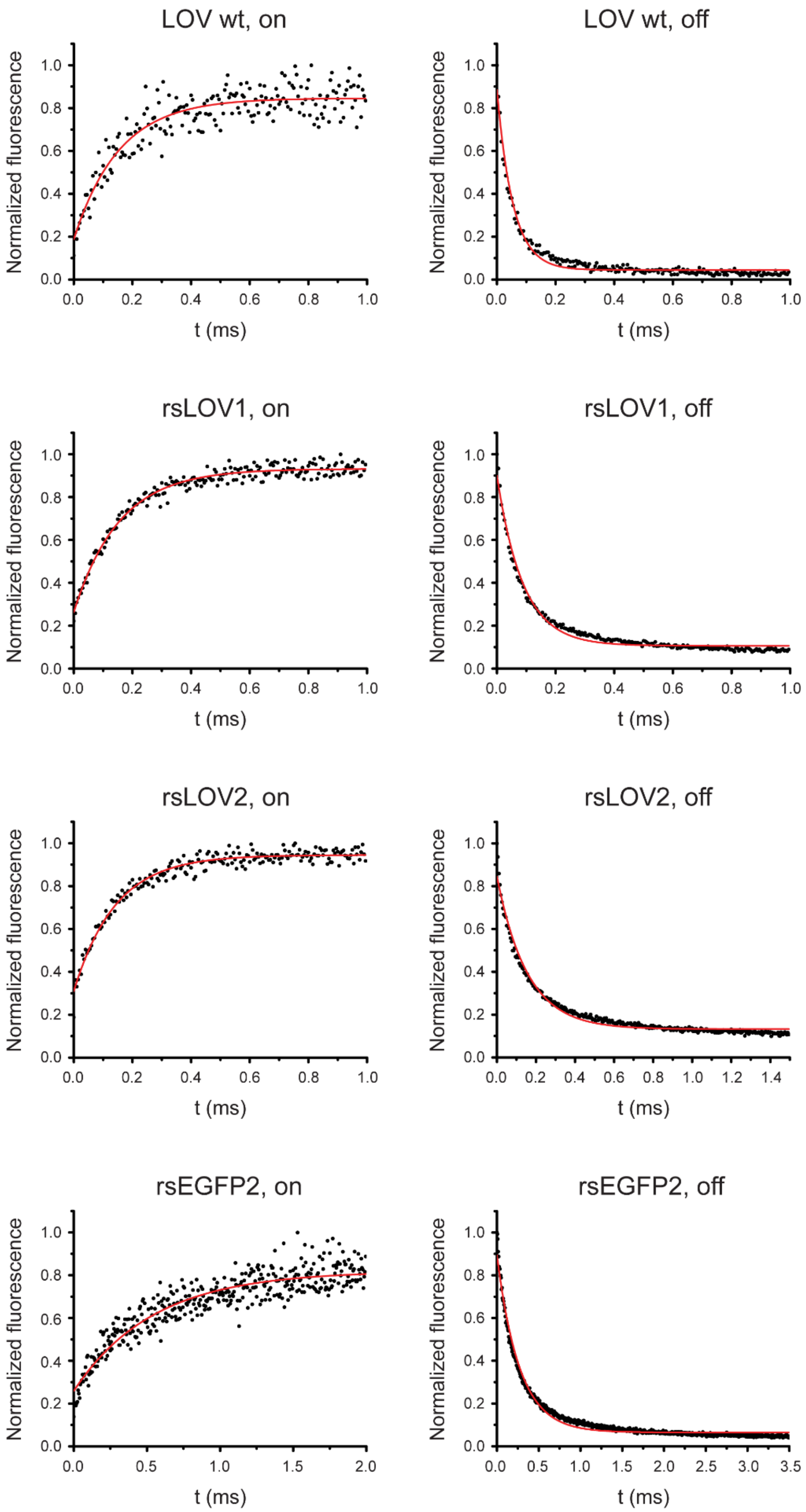

Supplementary Figure S3. Curve fits of on- and off-switching of YtvA-LOV wt, rsLOV1, rsLOV2, and rsEGFP2. To determine the switching time constants displayed in Fig. 3, switching curves of purified proteins were fitted with a single exponential function (red). One of 15 analyzed switching curves is shown in each case.

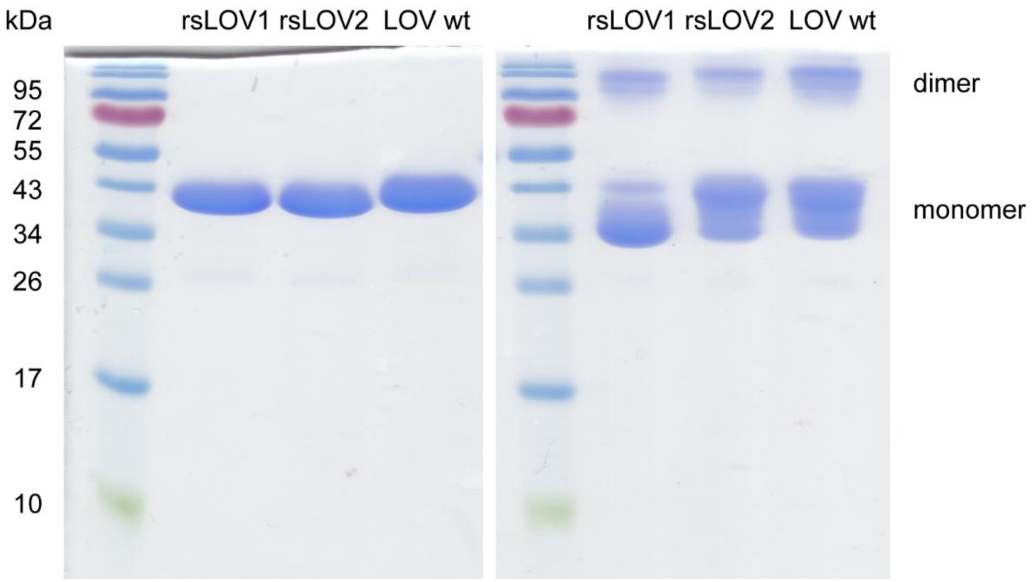

Supplementary Figure S4. Denaturing and seminative gel of GST-tagged rsLOV1, rsLOV2, and LOV wt. Proteins were purified from *E. coli* SURE cells. 15 µg of each protein was applied to a denaturing gel (left) or a seminative gel (right). Gels were stained with Coomassie Brilliant Blue.

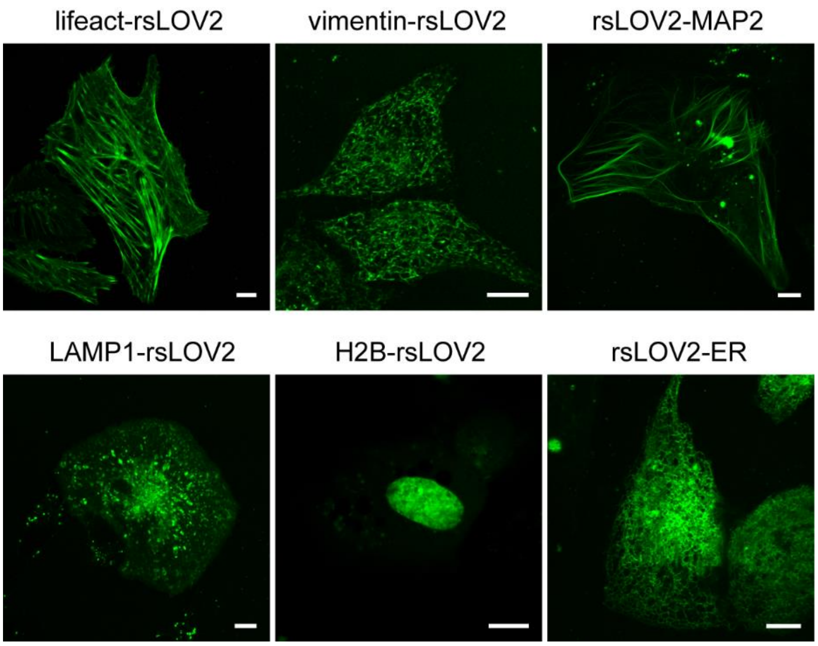

Supplementary Figure S5. Expression of different fusion proteins of rsLOV2 in living cells. Confocal images of lifeact-rsLOV2, rsLOV2-MAP2, LAMP1-rsLOV2, histone H2B-rsLOV2, and rsLOV2-KDEL for targeting the endoplasmic reticulum expressed in CV-1 cells. Vimentin-rsLOV2 was expressed in HeLa cells. Scale bars: 10 µm.

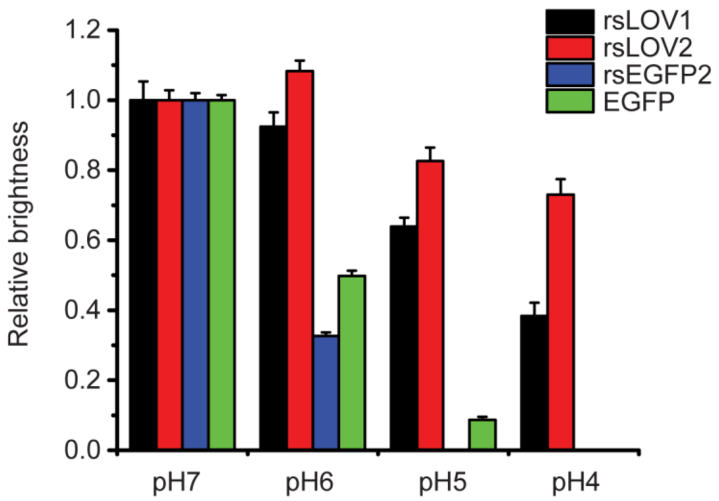

Supplementary Figure S6. Brightness of rsLOV1, rsLOV2, rsEGFP2, and EGFP at different pH values. The switching of purified proteins was measured in a citric acid/phosphate buffer at different pH values (off-switching: 488 nm, ~2 W·cm^{-2}, on-switching: 405 nm, ~5 W·cm^{-2}). The amplitudes of the off-switching curves fitted with a single exponential function (RESOLFT brightness) are compared. For EGFP, the brightness with 488 nm excitation is shown. Error bars represent standard deviation of the mean (n=10).

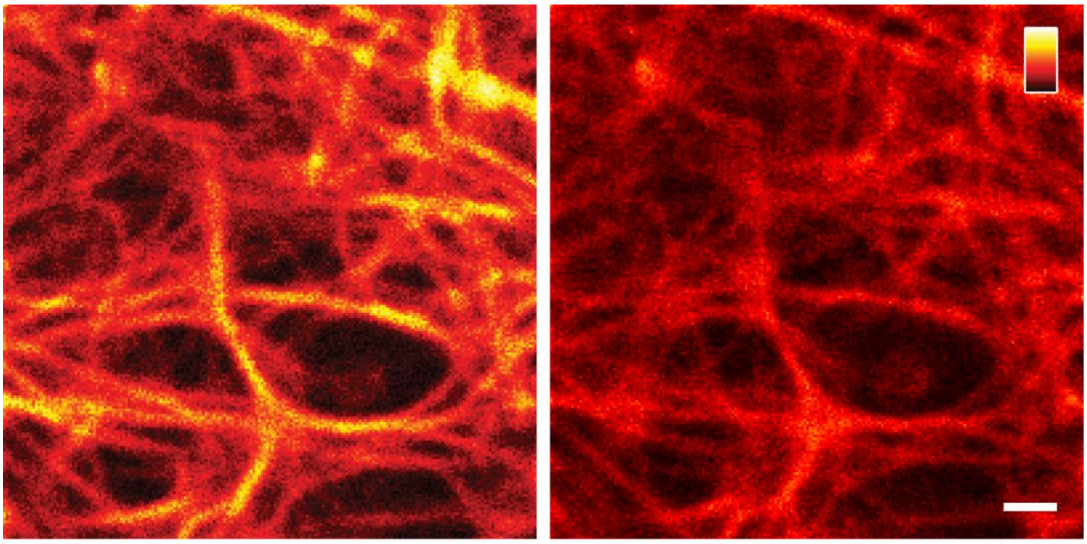

Supplementary Figure S7. Confocal images before and after STED imaging. CV-1 cells expressing lifeact-rsLOV2 were imaged confocally before (left) and after (right) STED imaging using 3 line scans with 488 nm excitation (3.0 µW, pulsed) and 618 nm STED (38 mW, pulsed) and a pixel dwell time of 200 µs. The same colormap was used for both images. 64% percent of the confocal signal is retained after STED imaging. Scale bar: 1 µm.
